# Application of dynamic metabolic flux analysis for process modeling: Robust flux estimation with regularization, confidence bounds, and selection of elementary modes

**DOI:** 10.1002/bit.27340

**Published:** 2020-05-12

**Authors:** Lukas Hebing, Tobias Neymann, Sebastian Engell

**Affiliations:** ^1^ Bayer AG Leverkusen Germany; ^2^ Process Dynamics and Operations group TU Dortmund University Dortmund Germany

**Keywords:** CHO fermentations, dynamic metabolic flux analysis, elementary modes, process modeling

## Abstract

In macroscopic dynamic models of fermentation processes, elementary modes (EM) derived from metabolic networks are often used to describe the reaction stoichiometry in a simplified manner and to build predictive models by parameterizing kinetic rate equations for the EM. In this procedure, the selection of a set of EM is a key step which is followed by an estimation of their reaction rates and of the associated confidence bounds. In this paper, we present a method for the computation of reaction rates of cellular reactions and EM as well as an algorithm for the selection of EM for process modeling. The method is based on the dynamic metabolic flux analysis (DMFA) proposed by Leighty and Antoniewicz (2011, *Metab Eng*, 13(6), 745–755) with additional constraints, regularization and analysis of uncertainty. Instead of using estimated uptake or secretion rates, concentration measurements are used directly to avoid an amplification of measurement errors by numerical differentiation. It is shown that the regularized DMFA for EM method is significantly more robust against measurement noise than methods using estimated rates. The confidence intervals for the estimated reaction rates are obtained by bootstrapping. For the selection of a set of EM for a given st oichiometric model, the DMFA for EM method is combined with a multiobjective genetic algorithm. The method is applied to real data from a CHO fed‐batch process. From measurements of six fed‐batch experiments, 10 EM were identified as the smallest subset of EM based upon which the data can be described sufficiently accurately by a dynamic model. The estimated EM reaction rates and their confidence intervals at different process conditions provide useful information for the kinetic modeling and subsequent process optimization.

## Graphical abstract

A method for the robust estimation of reaction rates in bioprocesses from concentration measurements is presented.This method is used in a framework for the generation of process models in which Elementary Modes (EM) of metabolic network are used as reactions. An algorithm for the selection of EM ensures, that the model complexity is kept as low as possible. The concept is applied to data of a CHO fed‐batch fermentation.

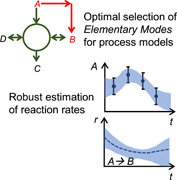

## 1 INTRODUCTION

For model‐based optimization of fermentation processes, for example, for process design or control, simple dynamic models which are accurate enough to predict the process behavior under varying conditions are needed (Frahm et al., 2002; Neddermeyer, Rossner, & King, 2015; Teixeira, Alves, Alves, Carrondo, & Oliveira, 2007).

Essential elements of models of fermentation processes are the stoichiometry of the biochemical conversion and the dependency of the reaction rates on the process conditions. The metabolism of the cells is very complex and comprises hundreds of chemical reactions, so that it is infeasible to derive rate equations for all these reactions. For the derivation of efficient models—efficient meaning sufficiently accurate with predictive capabilities but not overly complex—the usage of small metabolic networks at steady state (Nolan & Lee, 2011) or selections of elementary modes (EM) as macro reactions (Gao, Gorenflo, Scharer, & Budman, 2007; Provost, 2006; Soons, Ferreira, & Rocha, 2011; Teixeira et al., 2007) have been shown to be a powerful approaches. EM are calculated from a metabolic network and therefore provide a physiologically meaningful abstraction of the metabolism without the need of including dynamic intracellular mass balances and reaction kinetics. Formal kinetics or black‐box models like multilayer perceptron networks (MLP) can then be used to model the dependency of the reaction rates of the EM on the process conditions or on the concentrations of species in the reactor.

Alternative modeling approaches use empirical qualitative reaction schemes as macro reactions and fit the corresponding stoichiometric coefficients to data (Herold & King, 2014; Mailier & Wouwer, 2009). The complexity of these models is comparable with the complexity of models which use EM as macro reactions, as internal balances and reactions are lumped onto a few macroscopic pathways. However, physiological constraints as, for example, balances of internal components, energy carriers, or redox‐species cannot be taken into account and available biological knowledge is neglected.

For the generation of models that are based on EM it is necessary to (a) select a set of EM from a usually large number of possible EM and (b) select and fit kinetics for each of the reactions in the set.

Previously published methods for the selection of EM use estimated uptake or secretion rates. Soons et al. (2010) use a controlled random search algorithm to find the best set of EM which minimizes the difference to the estimated rates, and Abbate et al. (2019) link the number of reactions to the fraction of the explained variance in the estimated rates by comparing eigenvalues of a SVD decomposition. The subset is then found by solving a linear optimization problem. In both approaches, the trade‐off between the size of the set of EM and the accuracy of the representation of the estimated rates is exploited.

For the selection and fitting of kinetics, estimates of the cell‐specific EM reaction rates are needed. Several algorithms that have been proposed for this analysis use measured cell‐specific fluxes of medium components (Poolman, Venkatesh, Pidcock, & Fell, 2004; Schwartz & Kanehisa, 2006).

A disadvantage of these algorithms for the selection of the EM and the estimation of their reaction rates is that the estimation of the cell‐specific uptake or secretion rates has to be carried out first. This implies that derivatives of concentrations have to be computed in the first step, and, as the data is usually significantly corrupted by measurement errors, the resulting rates show large fluctuations as the derivation amplifies the errors.

In this paper, we present methods for the analysis and selection of EM for process modeling where measurement data is used directly: A method for the analysis of EM reaction rates is presented which is based on the approach for dynamic metabolic flux analysis (DMFA) by Leighty and Antoniewicz (2011) for the computation of internal flux distributions of a metabolic network. This method was not developed for the analysis of EM, but, as will be shown, it can be used for this purpose as well. The advantage of the approach by Leighty and Antoniewicz (2011) is that random noise in the measurements is attenuated by solving a linear optimization problem such that smoothing and numerical differentiation is not necessary. The method from Leighty and Antoniewicz (2011) is extended in this paper by the following elements:

- Additional linear constraints are included so that the fluxes are estimated taking into account the irreversibility of certain reactions.
- For large sets of reactions, the objective which was proposed in the approach of Schwartz and Kanehisa (2005) is considered in the objective of the DMFA method as a regularization term.
- The propagation of the measurement errors to the computed rates is obtained by bootstrapping.

It is shown that the regularized DMFA for EM method is more robust against measurement noise than other methods which are based upon the estimation of cell specific fluxes and that tuning of the algorithm is straightforward by using cross‐validation.

The selection of a subset of EM which are suitable for dynamic process modeling is determined by the following procedure:

(1) Before the EM are analyzed, the original DMFA problem with additional constraints to account for the irreversibility of certain reactions provides the best possible fit for a given metabolic network and therefore can be used as a benchmark. A suitable selection of EM should exhibit a comparable fit to the data. If the quality‐of‐fit is sufficient, the selection of EM can be carried out, otherwise essential reactions in the metabolic network are missing.
(2) A first reduction is carried out by means of *geometrical reduction* in which geometrically similar EM are discarded from the possibly large set of possible EM.
(3) For a further reduction, a multiobjective genetic algorithm (GA) is used together with the *DMFA for EM* method. The two objectives of the multiobjective GA are to optimize the fit of the predictions to the measured data and to minimize the size of the employed subset of the EM To explore the trade‐off between a good fit to the data and a low number of EM which is preferable because it reduces the model complexity.

**Figure 1:**
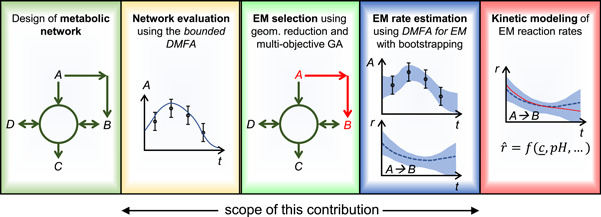
Overview of the steps for the selection and analysis of EM for process modeling. After a metabolic network has been chosen, the possible quality‐of‐fit can be tested using the bounded DMFA method. If this fit is sufficient, a selection of EM from this network can be performed using the geometrical reduction and the multiobjective genetic algorithm. The reaction rates of the selected set of EM and their confidence intervals are then obtained from the DMFA for EM method with bootstrapping. To obtain a dynamic model, kinetic equations are finally fitted to the estimates of the reaction rates. DMFA, dynamic metabolic flux analysis; EM, elementary modes [Color figure can be viewed at wileyonlinelibrary.com]

After a set of EM has been found, the pdf of the corresponding EM reaction rates over time at different process conditions can be evaluated by a bootstrap method in which the estimation is repeated with resampled measurements. This information is useful for selecting and fitting kinetic expressions of the reactions by statistical methods. A quantification of the uncertainty of the estimated cell‐specific rates is necessary as the magnitude of this uncertainty varies considerably during the course of a fermentation process. Figure 1 gives a graphical overview about the different steps of our modeling procedure.

Some elements of this procedure were already published presented in (Hebing, Neymann, Thüte, Jockwer, & Engell, 2016). This contribution extends this study by (a) adding regularization to the *DMFA for EM* problem, (b) adding linear constraints to the original DMFA approach, (c) showing how the *bounded DMFA* method can be used for the evaluation of a metabolic network, and (d) calculating confidence intervals of the specific reaction rates.

The selection and analysis of EM from a small metabolic network is demonstrated in Section 4 with real data from a CHO cultivation fed‐batch process under varying conditions based on an extended metabolic network from Nolan and Lee (2011). The selection and fitting of the kinetic equations based on these estimates is only sketched, as this is beyond the scope of this contribution.

## 2 THEORY

In this section, a short introduction of the original DMFA method by Leighty and Antoniewicz (2011) (which we will call *unbounded DMFA*) and the related new formulations (*bounded DMFA* and *DMFA for EM*) is given. Furthermore, the evaluation of the confidence intervals of the DMFA estimates and the regularization are explained.

### 2.1 Dynamic metabolic flux analysis

The differential equations that govern the evolution of the concentrations of the species in the reaction medium in a batch process (i.e., without addition or removal of substances from the reaction volume) can be calculated from the time‐dependent vector of fluxes in the metabolic network, $\underline{\nu }(t)$:

(1) $$\frac{d\underline{c}}{dt}=P\;\cdot \;\underline{\nu }(t)\;\cdot \;X_{v}(t),$$

where *P* is the (external) stoichiometric matrix, $\underline{\nu }(t)$ the cell‐specific flux vector, and $X_{v}\left(t\right)$ is the concentration of viable cells. The volumetric flux vector $\underline{V}\left(t\right)$ is:

(2) $$\underline{V}(t)=\underline{\nu }(t)\;\cdot \;X_{v}(t).$$

The evolution of the concentrations of the species inside the cell can also be calculated from the time‐dependent fluxes in the metabolic network, $\underline{\nu }(t)$. The *steady state* assumption for the internal metabolites in metabolic flux analysis (MFA) postulates that the derivatives of the internal concentrations are zero. This results in a, usually under‐determined, system of linear equations:

(3) $$N\;\cdot \;\underline{V}(t)=0,$$

where *N* is the (internal) stoichiometric matrix. Under this assumption, $\underline{V}\left(t\right)$ can be calculated from volumetric rates of the so‐called free fluxes $\underline{U}\left(t\right)$:

(4a) $$\underline{V}(t)=K\;\cdot \;\underline{U}(t),$$

(4b) K=null(N),

where null*(N)* denotes that N⋅K=0. All fluxes which satisfy Equation ((4a), (4b)) fulfill the *steady state* assumption for the internal metabolites.

Leighty and Antoniewicz (2011) assume that the *volumetric* free fluxes $\underline{U}\left(t\right)$ are piece‐wise linear over time between inflection points $T_{j}$, such that Equation (1) has a simple analytic solution. The values of $U_{k}\left(T_{j}\right)$ can then be determined by solving the *unbounded* DMFA problem (5):

(5) $$\min_{U_{1}(T_{1}),\;U_{1}(T_{2})\text{…}.}SSR,$$

where sums of squared residuals (SSR) is the sim of squared residuals:

(6) $$SSR=\sum_{i=1}^{n_{c}}\sum_{j=1}^{n_{t}}{\left(\frac{c_{i}^{m}\left(t_{j}\right)-{\hat{c}}_{i}\left(t_{j}\right)}{\sigma_{i}\left(t_{j}\right)}\right)}^{2}.$$

Each ${\hat{c}}_{i}(t_{j})$ in Equation (6) can be computed as a linear combination of the estimated fluxes $U_{k}(T_{j})$. The number of inflection points is usually chosen manually according to the expected profiles of the volumetric rates which are computed by this method.

In general, the resulting estimation problem is over‐determined and the estimated concentration profiles ${\hat{c}}_{i}(t_{j})$ therefore are smoothened and the influence of the measurement noise is averaged out to a certain extent. The smoothing of the estimated concentration profile is a consequence of the limited flexibility of the assumed piece‐wise linear volumetric reaction rates. For further details, the reader is referred to Leighty and Antoniewicz (2011).

In the bounded DMFA problem, constraints for certain internal fluxes that are non‐negative due to the irreversibility of the corresponding reactions ${\underline{V}}_{\mathrm{irr}}$ are taken into account:

(7a) $$\min_{U_{1}(T_{1}),U_{1}(T_{2})\text{…}.}SSR.$$

*s.t*.

(7b) $${\underline{V}}_{irr}(T_{j})=K_{Irr}\;\cdot \;\underline{U}(T_{j})\geq 0.$$

All fluxes which are calculated from Equations ((4a), (4b)) and ((7a), (7b)7) fulfill the *steady state* assumption and the irreversibility constraints.

The (volumetric) fluxes $\underline{V}\left(t\right)$ which fulfill the *steady state* assumption and irreversibility constraints can also be expressed as a non‐negative linear combination of reaction rates of EM, assembled in the vector $\underline{R}\left(t\right)$ (Schuster & Hilgetag, 1994):

(8) $$\underline{V}\left(t\right)=E\cdot \underline{R}\left(t\right),$$

where *E* is the *elementary mode matrix*. The profile of the (volumetric) EM reaction rates over time $\underline{R}\left(t\right)$ is obtained by solving the *DMFA for EM* problem (9):

(9a) $$\min_{R(T_{1}),\;R_{1}(T_{2})\text{…}.}SSR.$$

*s.t*.

(9b) $$R_{k}\left(T_{j}\right)\geq 0,\forall k\in \left\{1,\text{…},n_{r}\right\},\forall j\in \{1,\text{…},n_{T}\},$$

where $R_{k}(T_{j})$ is the value of the (volumetric) reaction rate of EM *k* at the inflection point $T_{j}$, $n_{r}$ is the number of EM and $n_{T}$ the number of inflection points. Each ${\hat{c}}_{i}(t_{j})$ can be computed as a linear combination of all estimated quantities $R_{k}(T_{j})$. The cell‐specific reaction rates $\underline{r}\left(t\right)$ can be obtained from the volumetric rates $\underline{R}\left(t\right)$ by:

(10) $$r_{k}\left(t\right)=\frac{R_{k}\left(t\right)}{X_{v}\left(t\right)}.$$

### 2.2 Regularization: Minimizing the norm of the specific reaction rates

For large sets of EM, the solution of the estimation problem (9) may lead to ill‐conditioned problems, so that even large changes in $R_{k}\left(T_{j}\right)$ result in only small changes of the value of the objective function, as many EM with a similar stoichiometry exits. To overcome this problem, a penalty term is added to the cost function:

(11) $$\min_{R\left(T_{1}\right),R_{1}\left(T_{2}\right)\text{…}.}\mathrm{SSR}+\alpha \;\cdot \;\sum_{k}\sum_{j}{\left(\frac{R_{k}\left(T_{j}\right)}{X_{v}\left(T_{j}\right)}\right)}^{2},$$

where $X_{v}$ is the concentration of viable cells and α is a scalar weighting factor which optimal value is found by cross‐validation. With this additional term, the L2‐norm of the cell‐specific rates is penalized. Penalizing cell‐specific rates is preferred over penalizing volumetric rates as unwanted and unrealistic behavior on the cellular level is less likely to occur. Otherwise, unrealistically high cell‐specific rates might be obtained from the *DMFA for EM* method in the beginning or at the end of a process where the concentration of viable cells is low. This criterion is also used by Schwartz and Kanehisa (2005) for the estimation of EM reaction rates.

### 2.3 Confidence intervals by resampling of measurements

Classical methods for the estimation of confidence intervals like the Cramer‐Rao lower bound are problematic in systems with many parameters from which a few might be unobservable, which can happen in the DMFA method when a large number of reactions are involved. Additionally, constraints on the reaction rates cannot be taken into account.

We therefore propose to use a bootstrap method instead. Here, the measurements are resampled from their expected probability density functions. Often, the probability density functions of the measurements can be assumed to be Gaussian distributed and the variances can be estimated or are available from sensor data. Alternatively, these uncertainties can be estimated by a maximum likelihood estimator.

The estimation of the reaction rates with the DMFA method can then be repeated using the sampled data. However, two major changes have to be made to get rid of the *estimation bias* by the DMFA method:

- The position of the inflection time‐points $T_{j}$ must be randomized.
- The regularization coefficient α must be zero or small such that numerically ill‐conditioned estimations can be carried out without strongly influencing the result.

From the sampled estimates of the reaction rates and their confidence intervals are obtained.

### 2.4 Choice of a subset of EM

The different variants of the DMFA method can be used to identify a set of active EM directly from measurement data and thus enable the modeler to select a suitable subset of EM for a dynamic process model.

The two DMFA problems *bounded DMFA* and *DMFA for EM* for the complete set of EM are both based on the *steady state* assumption for the internal metabolites and take the irreversibility of reactions into account. The minimum *SSR*, which can be calculated from Equations (7) and (9) therefore are equal if the complete set of EM is used. When some EM are removed from the complete set, the corresponding columns in *E* and elements of *R* (Equation (8)) are deleted and the calculated *SSR* will increase. So the *SSR* value from the solution of the *bounded DMFA* provides a lower bound which is only dependent on the assumed metabolic network. Choosing a subset of EM will lead to a higher *SSR* value, that is, a worse fit to the measurement data. If the results from the *bounded DMFA* are not sufficiently accurate, a modification of the metabolic network should be considered before a subset of EM is chosen.

For the selection of a subset for a process model, two algorithms are proposed:

(1) A geometrical reduction, which discards similar EM from the original set based on their cosine similarity. This algorithm can be used for a preliminary reduction if the initial number of EM is very large. The SSR value which is calculated using the *DMFA for EM* method can be used to ensure that no significant EM is removed. The algorithm is described in the appendix.
(2) A *multiobjective GA* which considers as objectives the *SSR* value and the number of EM in the set.

The degrees of freedom which the GA optimizes are binary decision variables $\xi_{i}$ for each EM. The variable $\xi_{i}$ determines whether the corresponding EM is part of the subset or not. For each selected subset, problem (9) is solved using a linear solver to obtain the corresponding *SSR* value. The optimization problem can be written formally as:

(12) $$\min_{\underline{\xi }}\left\{\mathrm{SSR},\sum \xi_{i}\right\}.$$

The resulting Pareto front describes the *SSR* which results from an optimized selection of EM as a function of the cardinality of the set of EM. The Pareto front helps the modeler to decide how many EM are necessary to capture the measured behavior of the process with an acceptable model error. The *SSR* value approaches the *SSR* value of the *bounded DMFA* problem when the number of EM in the subset increases.

Thus, before any kinetic expression is fitted, the *SSR* value of the resulting sets of EM give an indication of the expected quality‐of‐fit and of the complexity of the process model which is based on this set of reactions. Practically, one will search for the lowest number of EM which still provide a desired fit to the data.

## 3 SIMULATION STUDY: EM ANALYSIS USING NOISY CONCENTRATION DATA

In this simulation study, the capability of predicting reaction rates of EM from noisy concentration measurements is tested. We compare the *DMFA for EM* method that was presented above with other commonly used techniques that are based on the analysis of cell‐specific uptake or secretion rates, namely Poolman et al. (2004) and Soons et al. (2011). As the estimation method of these cell‐specific rates from time‐series of noisy concentration measurements plays a significant role in the analysis, several combinations for smoothing, interpolation, and filtering are also tested.

A small metabolic network with known kinetic terms was used to generate artificial measurement data of a fed‐batch process at different levels of measurement noise. The complete model, the chosen boundary conditions and the estimation methods are described in the appendix.

**Figure 2:**
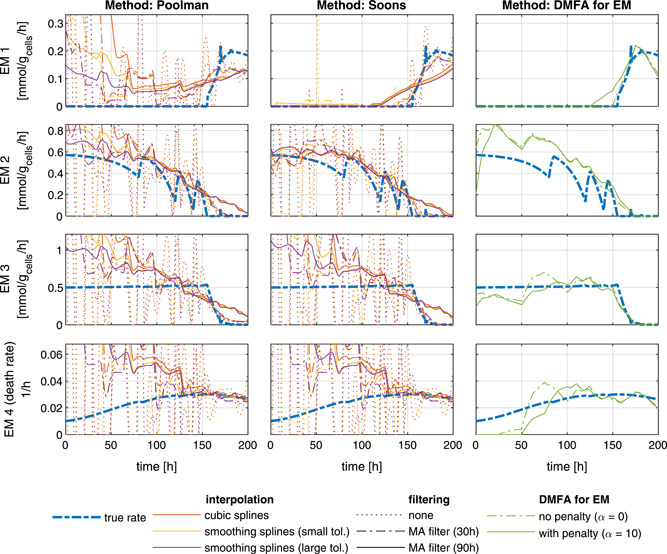
True reaction rates of all EM and estimations with the Poolman, Soons and DMFA for EM methods with interpolation and filtering steps. For this example, the variance of the Gaussian noise was chosen such that the 95% confidence interval equals 5% of the magnitude of the measurements. DMFA, dynamic metabolic flux analysis; EM, elementary modes [Color figure can be viewed at wileyonlinelibrary.com]

Figure 2 shows all estimated reaction rates together with the real reaction rates of the EM.

It can be seen that either smoothing or filtering is necessary to obtain good estimates when the Poolman or the Soons methods are used. The choice of the interpolation and filtering method which is used for calculating the specific rates has a significantly higher impact on the result than the estimation method itself. Table 1 shows the average approximation error at different levels of measurement noise. It can be seen that the estimations from the regularized *DMFA for EM* method are more accurate in the presence of realistic measurement noise. However, in the absence of noise the estimation is worse due to the lower flexibility of the piece‐wise linear volumetric rates.

**Table 1:** Average reconstruction error $\left|\hat{r}-r^{\mathrm{true}}\right|$ of different estimation methods for EM reaction rates $\hat{r}$ at different levels of simulated measurement noise

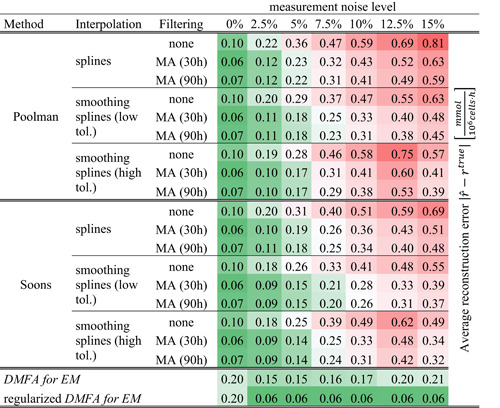

*Note*: The calculation of specific rates is carried out using different interpolation (splines, smoothing splines)‐ and filtering methods (MA = *moving average filter*). No smoothing and filtering is employed for the *DMFA for EM* method. More details are described in the appendix.

## 4 REAL WORLD EXAMPLE: EM SELECTION AND ANALYSIS USING EXPERIMENTAL CHO FED‐BATCH FERMENTATION DATA

The concept for the choice of an EM reaction set was applied to data‐sets from six fed‐batch fermentations of a CHO culture. One experiment was excluded from this procedure and used as a validation data set for the model which was built using the chosen set of EM and fitted to the other five data sets. For brevity, we will show only three of the remaining five experiments in the following which represent the most “extreme” responses of the process to different set‐points for the pH value and glucose levels. Measurements of the viable cell density ($X_{v}$), the total cell density, concentrations of the components of the medium, and dissolved oxygen‐sensor information were available and used. For confidentiality reasons, not more details can be given and not all measured components can be shown in the following.

### 4.1 Metabolic network

For the calculation of pseudo‐batch data from the fed‐batch measurements, the influence of the liquid feed as well as the mass‐transfer over the gas‐liquid phase boundary were compensated according to the *shifting* method which is described in the appendix.

The metabolic network from which the EM were calculated was taken from Nolan and Lee (2011) and extended by the glyoxylate‐cycle and a maintenance reaction in which ATP is consumed. The network reactions are listed in Table 2.

**Table 2:** Reactions of the reduced metabolic network from Nolan and Lee (2011), extended as described. Components which are written in red are extracellular

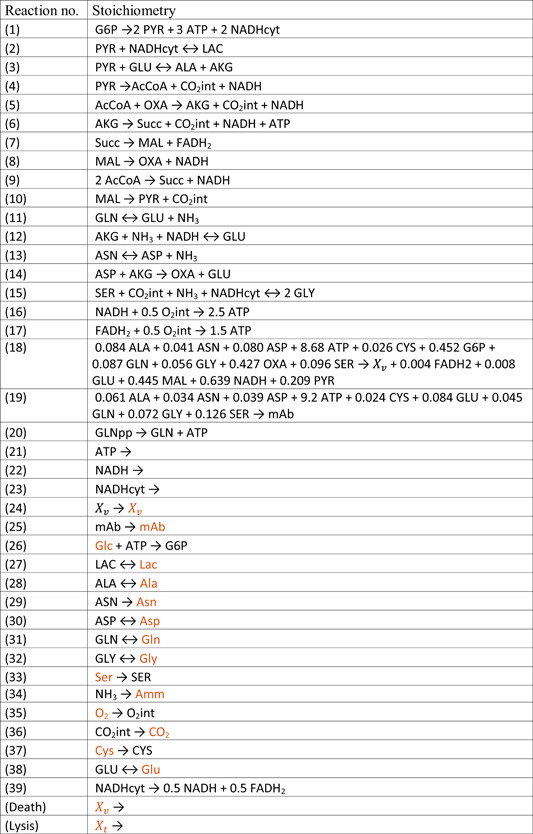

*Note*: Components which are written in red are extracellular.

Before the EM analysis and the choice of an EM subset, it was determined how well the network can explain the evolutions of the concentrations in the measured data. With the methods *unbounded DMFA* (5) and *bounded DMFA* (7), the fluxes of the reactions in the network and their confidence intervals were estimated by minimizing the difference between the estimated concentration profile and the (resampled) measured data (cf. Section 2.3).

**Figure 3:**
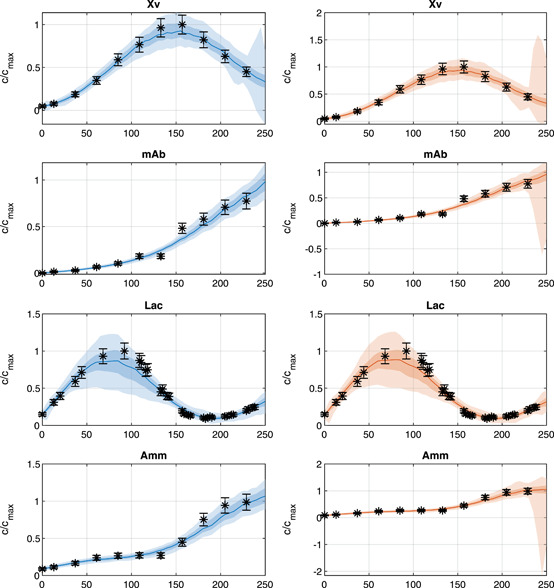
Measured data of different medium concentrations of one data set with the profiles of the estimated concentrations $\hat{c}$ which were generated using the *bounded DMFA* (in blue, left column) and *unbounded DMFA* (in red, right column) methods. The color shadings refer to the 95% and the 68% confidence bounds and the median of all estimations after 500 resamples of the measurements (cf. Section 2.3). The estimated evolutions of the concentrations do not differ much, but from the evolution of mAb and Amm it can be seen that flux bounds bounds are useful for the reduction of the confidence bounds as the uptake of these substances is ruled out in the *bounded DMFA*. DMFA, dynamic metabolic flux analysis [Color figure can be viewed at wileyonlinelibrary.com]

**Figure 4:**
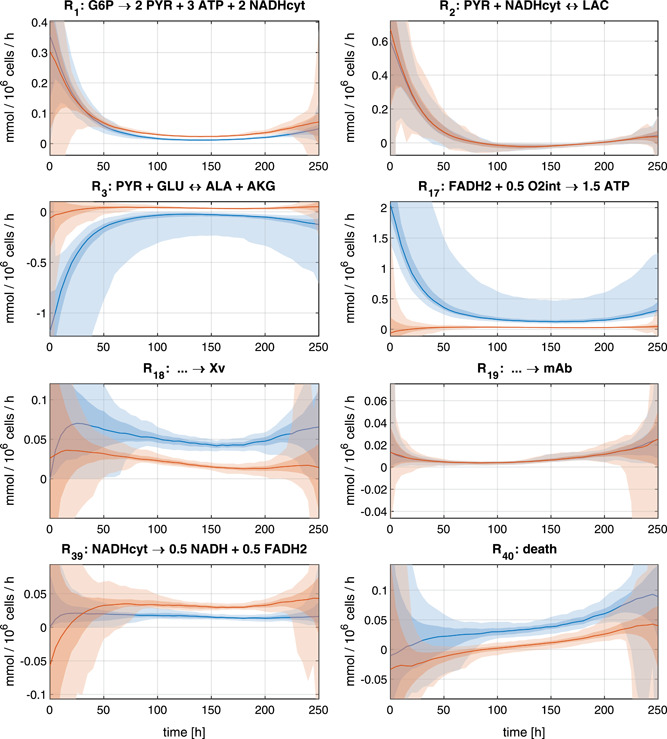
Estimated reaction rates of eight chosen reactions from the metabolic network obtained using the *bounded DMFA* (in blue) and *unbounded DMFA* (in red) methods for the data of one CHO fermentation fed‐batch experiment. The color shadings refer to the 95% and the 68% confidence bounds and the median of all estimations after 500 resamples of the measurements (cf. Section 2.3). Although the estimated concentrations $\hat{c}$ are similar in the *unbounded* and *bounded* estimations, the differences in the estimated rates are quite large for some intracellular reactions. The major reason for this is the reversibility of the death rate in the *unbounded DMFA* method which also leads to lower reaction rates in anaplerotic reactions in the TCA cycle. The low concentration of the biomass $X_{v}$ in the beginning of the process leads to larger confidence bounds of the rates at these time‐points. DMFA, dynamic metabolic flux analysis [Color figure can be viewed at wileyonlinelibrary.com]

Figure 3 shows the fit to the data of one experiment for four out of 10 different concentration profiles. Figure 4 shows the estimated reaction rates from eight chosen reactions of the metabolic network. It can be seen that the irreversibility of some reactions is violated when the unbounded DMFA method is used which leads to unrealistic results.

The quality‐of‐fit of the *bounded DMFA* method determines how well the measured concentration profiles can be expressed by any selection of EM of the network. As this quality‐of‐fit is satisfactory, the EM analysis and the selection of a subset of EM were carried out.

### 4.2 Identifying the set of active EM

**Figure 5:**
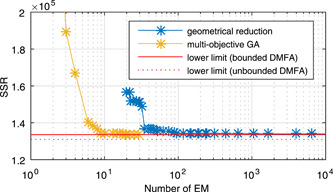
Pareto front of the size of the optimized EM subsets and the corresponding optimized *SSR* values. The red lines indicate the *SSR* values which were obtained by solving the *unbounded*‐ and *bounded* DMFA problems. The SSR values for different EM subsets approaches the *SSR* value of the *bounded DMFA* with an increasing number of EM in the set. DMFA, dynamic metabolic flux analysis; EM, elementary modes; SSR, sums of squared residuals [Color figure can be viewed at wileyonlinelibrary.com]

Using the *metatool*‐software (Pfeiffer, Nu, Montero, & Schuster, 1999), more than 18,000 EM were calculated from the network. With the geometrical reduction technique, based on the cosine similarity (cf. chapter 2.4), this number was first reduced. The resulting approximation errors for the different EM selections are shown in Figure 5. The tolerance value in the geometrical reduction was set to 1% of the initial *SSR*. With <100 EM this tolerance value is exceeded. It can be seen that a further reduction of the set of EM below 100 would lead to an significant increase of the *SSR* when the geometrical reduction is used.

The multiobjective GA, described in Section 2.4, was then used to generate optimal selections of EM on the Pareto front between the approximation quality (*SSR*) over the number of EM. In each evaluation of the objective function of the multiobjective GA, the *DMFA for EM* method was used for all experiments. The number of inflection points was set to 5. Figure 5 shows the calculated *SSR* values for different reduced sets of EM together with the *SSR* of the *unbounded* and *bounded DMFA* problem for the training data. In this study, the MATLAB® implementation *gamultiobj* was used in which the NSGA‐II algorithm is utilized. Custom mutation and crossover functions were created to account for the binary variables $\xi_{i}$ (Equation (12)). The computation time of the GA was approximately 4 hours on an Intel four core i7 desktop computer with 2.67 GHz.

The *SSR* value of the *bounded DMFA* problem provides the lower limit of the *SSR* values for the subsets of EM. This lower bound is only dependent on the network itself and not on the number and the choice of the EM. The geometrical reduction turned out to be a suitable algorithm for reducing large sets of EM without a loss of physiologically important EM, but if smaller subset sizes are aimed at, the evolutionary algorithm gives much better results.

**Figure 6:**
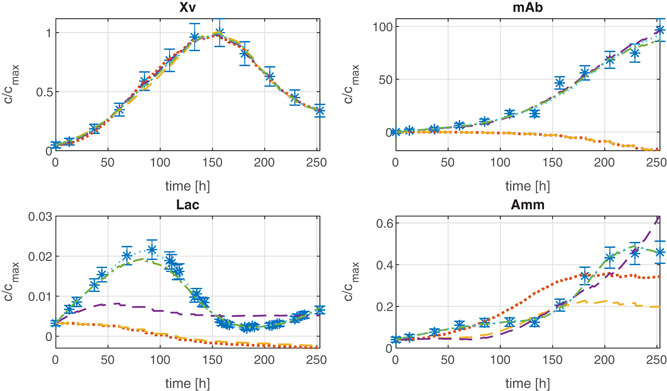
Measured data of four different medium concentrations of one experiment of the CHO fermentation with the profiles of the estimated concentrations *ĉ* which were generated by the *DMFA for EM* method using different EM subsets from the Pareto front of Figure 5. The sizes of the subsets are: 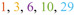. Additionally, the death‐rate was also included. DMFA, dynamic metabolic flux analysis; EM, elementary modes [Color figure can be viewed at wileyonlinelibrary.com]

From the Pareto front of the multiobjective evolutionary algorithm, it is easily possible to select a subset of EM for a process model. The set should be as small as possible but with an acceptable *SSR* value (i.e., an acceptable fit to the data). The fit to the data of one experiment for four out of ten different concentration profiles is shown for different selections of EM subsets in Figure 6. As expected from the Pareto front (cf. Figure 5), the quality of fit of subsets with 10 reactions and more is comparable to the result obtained by the *bounded DMFA* method. For <10 reactions, the approximation becomes significantly worse. With <10 EM, essential pathways seem to be missing and the data cannot be reconstructed well enough. The stoichiometry of the 10 EM is shown in Table 3. The actual state of the metabolism can sufficiently be described by a combination of these metabolic modes. The following metabolic features are present in the set of EM:

- Oxic‐ and anoxic conversion of glucose with‐ and without lactate formation
- Different yields for biomass and/or product formation
- Utilization of different nitrogen sources
- Reutilization of by‐products like lactate.

**Table 3:** Stoichiometry of the chosen subset of EM consisting of 10 EM

*EM 1*:	0.1649Glc + 0.010215Lac + 0.022254Gln + 0.66661O_2_→0.36483$X_{v}$ + 0.062105Amm + 0.50773CO_2_
*EM 2*:	0.47847Glc + 0.006275Gln + 0.015173O_2_→0.10287$X_{v}$ + 0.86106Lac + 0.090627CO_2_
*EM 3*:	0.23253Glc + 0.0004173Gln + 9.2732e−05O_2_→0.046366$X_{v}$ + 0.42958Glu + 0.87011CO_2_
*EM 4*:	0.045706Glc + 0.41079Lac + 0.37711Gln + 0.059863Amm + 0.18476O_2_→0.10112$X_{v}$ + 0.79296CO_2_
*EM 5*:	0.26767Glc + 0.014165Gln + 0.72659Amm + 0.50654O_2_→0.31478mAb + 0.1456Glu + 0.15284CO_2_
*EM 6*:	0.58685Glc + 0.024585Gln + 0.57456O_2_→0.40304$X_{v}$
*EM 7*:	0.85201Glc + 0.01146Gln + 0.213O_2_→0.25468mAb + 0.40461Glu
*EM 8*:	0.065947Glc + 0.655Lac + 0.53142Glu + 0.012693Gln + 0.26659O_2_→0.1459$X_{v}$ + 0.41281CO_2_
*EM 9*:	0.70711Glu→0.70711Gln
*EM 10*:	0.57735Gln→0.57735Glu + 0.57735Amm
*Death rate*:	$X_{v}$→

*Note*: The death rate is added as an additional reaction.

Abbreviation: EM, elementary modes.

### 4.3 EM reaction rates and confidence intervals

**Figure 7:**
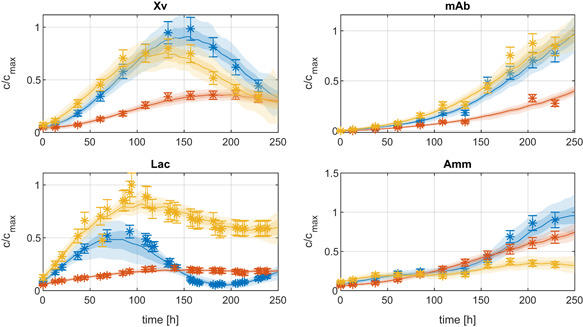
Concentration data of three different experiments at different conditions 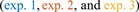 with the profiles of the estimated concentrations *ĉ* which were generated using the *DMFA for EM* method using the selected 10 EM. The color shadings refer to the 95% and the 68% confidence bounds and the median of all estimations after 500 re‐samples of the measurements (cf. section 2.3). DMFA, dynamic metabolic flux analysis; EM, elementary modes [Color figure can be viewed at wileyonlinelibrary.com]

**Figure 8:**
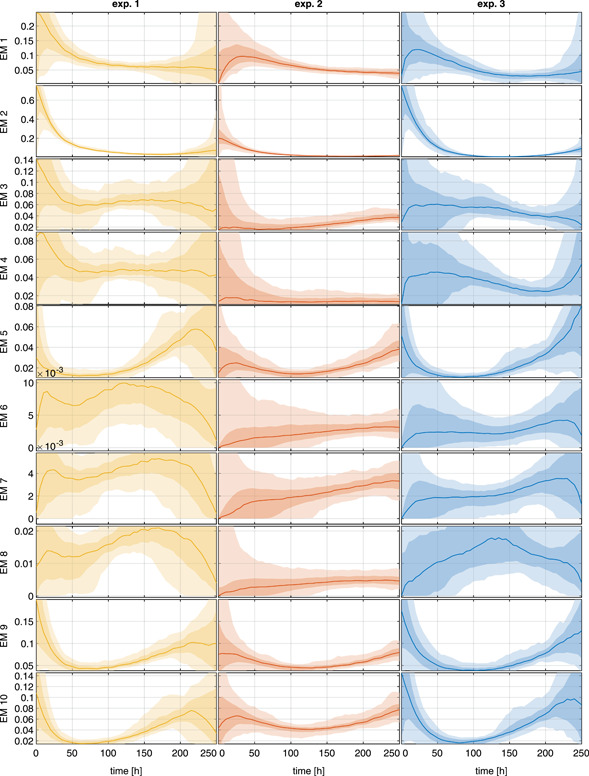
Estimated EM reaction rates *r*(*t*) [mmol/10^6^ cells/h] of three different experiments at different conditions 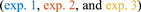 which were generated using the *DMFA for EM* method using the selected 10 EM. The color shadings refer to the 95% and the 68% confidence bounds and the median of all estimations after 500 re‐samples of the measurements (cf. section 2.3). DMFA, dynamic metabolic flux analysis; EM, elementary modes [Color figure can be viewed at wileyonlinelibrary.com]

After the set of 10 EM has been found, the confidence intervals for the EM reaction rates are estimated using the bootstrap method (cf. Section 2.3). For each component, the measurement uncertainty was assumed to be a linear function of the magnitude of the measurement:

(13) $$\sigma_{i}(t_{j})=a_{i}+b_{i}\;\cdot \;c_{i}(t_{j}),$$

where $a_{i}$ and $b_{i}$ were estimated from replicates of the experiments. The sampled estimates $\hat{c}$ for four different components in three different experiments are shown in (Figure 7). The differences in this experiments are due to different process conditions. The estimated EM reaction rates and their confidence intervalsare shown in Figure 8. It can be seen that the magnitude of the estimation uncertainty varies significantly over time. Especially in the beginning and at the end of the process, the confidence bounds of the reaction rates are very large. This is due to the low concentration of viable cells. As a consequence, no significant differences in the reaction rates can be observed. Only in the middle of the process, some reaction rates are clearly different due to the different process conditions. In this example, the reaction rates of $EM_{1}$, $EM_{5}$, and $EM_{9}$ are not significantly influenced by the different process conditions but the other EM show observable differences.

### 4.4 Further modeling steps

After the EM have been chosen and analyzed, kinetic equations must be selected for each reaction which should adequately represent the influence of the process conditions on the reactions. The selection and fitting of kinetics is not in the scope of this paper so these steps are only sketched.

The information about reaction rates and their confidence intervals is very useful for the modeling of the process, as it enables the modeler to differentiate between significant and nonsignificant influences on the reaction rates. The kinetic functions for the reaction rates $\hat{r}=f(\underline{c},\mathrm{pH},T,\text{…})$ should express only the significant influences.

The results of the real‐world example showed that the evaluation of the confidence intervals is especially useful for fitting specific rate equations to estimates as the observability of these rates heavily depends on the state of the process. In this example, the range of the confidence intervals comprises several orders of magnitude.

In an earlier contribution (Hebing et al., 2016; Neymann, Hebing, & Engell, 2019) we proposed to select and fit nonlinear reaction kinetics $\hat{r}\left(\Theta \right)$ to estimated rates of EM *r* by solving:

(14) $$\min_{\Theta }\sum_{i}{\left(\frac{\hat{r}\left(t_{i},\Theta \right)-r\left(t_{i}\right)}{\sigma \left(t_{i}\right)}\right)}^{2},$$

here, Θ is the parameter vector and $\sigma (t_{i})$ is the standard deviation of the estimate which can be obtained from the bootstrap samples of the estimate r(t). Only with a reliable estimate of σ, it is possible to fit and compare meaningful kinetics based on a statistical measure as, for example, the Akaike information criterion.

Also black‐box models like MLP for reaction kinetics of the selected EM can be fitted to the estimated reaction rates r(t), for example, by back‐propagation. Here also, the standard deviation of the rates σ(t) can be used for the weighting of the values at different time‐points. If this information is neglected, the identified rate expressions would be corrupted by unreliable estimation noise, especially in beginning and at the end of the fed‐batch process where the observability of the specific reaction rates is bad due to a low viable cell density.

With the selected EM and fitted kinetics, the dynamic process model for fermentation processes is ready to be used for process design, optimization, model‐predictive control, or other purposes.

## 5 CONCLUSION

This paper presents methods for the selection of small sets of EMs and for the estimation of reaction rates from noisy concentration measurements. We propose extensions to the method of Leighty and Antoniewicz (2011) which help to (a) consider irreversible reactions in the estimation and (b) increase the robustness against measurement noise due to additional regularization.

It could be shown in a simulation study that the DMFA for EM method is superior for estimating EM reaction rates from noisy concentrations measurements.

The presented algorithm for the selection of small subsets of EM was used to select a suitable set of EM for a model of a CHO fed‐batch fermentation using real‐world data. It could be shown that the metabolism can be described by 10 EM with an acceptable accuracy at the experimental conditions.

Using a bootstrap resampling method, the confidence intervals of the EM reaction rates of this set were calculated. This information can then be used for the choice and fitting of kinetic equations.

## APPENDIX A GEOMETRICAL REDUCTION

The geometrical reduction is based on the mutual cosine similarity $\Gamma_{i,j}$ of two EM ${\underline{e}}_{i}$ and ${\underline{e}}_{j}$ (which are columns of the EM matrix E, cf. Equation (8)):

(15) $$\Gamma_{i,j}=\frac{{\underline{e}}_{i}\;\cdot \;{\underline{e}}_{j}}{\left\|{\underline{e}}_{i}\right\|\;\cdot \;\left\|{\underline{e}}_{j}\right\|}.$$

The reduction is carried out in consecutive steps in which the estimation error of the reduced set $\text{SS}R^{\text{red}}$ increases. The complete algorithm is described in Algorithm 1.

Algorithm 1 

**Table:**

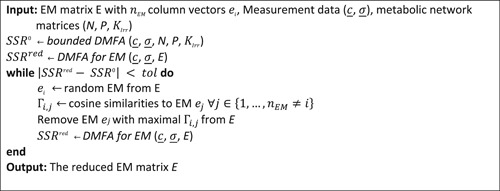

## APPENDIX B SHIFTING OF CONCENTRATIONS

The DMFA problems (5–11) are based on the analytical solution of the ODE, which describes the evolution of the concentrations in a batch process. To apply the DFMA approach to fed‐batch processes, *pseudo batch data* can be computed from the measured data of a fed‐batch process. The accumulated effects of all changes of measured concentrations Δc(t) which are caused by inputs to the process (i.e., feeds and the exchange of mass with the gas phase) and not by the metabolism of the cells are subtracted from the trajectories of the concentrations. The resulting concentration profiles are called *shifted* profiles and are computed according to:

(16a) $$c_{i,s}^{m}\left(t\right)=c_{i}^{m}\left(t\right)-\Delta c_{i}(t),$$

(16b) $$\Delta c_{i}\left(t\right)=\int_{0}^{t}\left[{\dot{c}}_{i,\mathrm{feed}}\left(\tau \right)+{\dot{c}}_{i,\mathrm{gas}}\left(\tau \right)\right]d\tau .$$

The resulting shifted measurements can be used as if they were batch data in the solution of the DMFA problems (5–11). The solution of the optimization problems (5–11) provides estimated shifted concentrations ${\hat{\underline{c}}}_{s}$.

## APPENDIX C GENERATION OF ARTIFICIAL FED‐BATCH DATA

In the simulation study, a fictive small metabolic network, consisting of three reactions, three external components and one internal component is used to generate synthetic data of a typical fed‐batch fermentation. A substrate *A* is converted to a intermediate product *B* which is then converted to viable biomass $X_{v}$ in reaction $\nu_{2}$ which has a limited capacity $\nu_{2,\max}$. If this limit is reached, the toxic external by‐product *C* is formed in a reversible reaction $\nu_{3}$. All reactions are catalyzed by the viable biomass $X_{v}$. It is assumed that the internal component *B* is neither consumed nor accumulated as the sum of the reaction rates $\nu_{2}$ and $\nu_{3}$ is always equal to $\nu_{1}$. The balance equations for the medium components are shown in Equations (17a)–(17m), the corresponding parameter values are listed in Table 4.

(17a) $$\frac{\mathrm{dA}}{\mathrm{dt}}=-\nu_{1}\;\cdot \;X_{v}+D\;\cdot \;(A_{\mathrm{in}}-A).$$

(17b) $$\frac{\mathrm{dC}}{\mathrm{dt}}=\nu_{3}\;\cdot \;X_{v}-D\;\cdot \;C.$$

(17c) $$\frac{dX_{v}}{\mathrm{dt}}=(\mu -\mu_{d})\;\cdot \;X_{v}-D\;\cdot \;X_{v}.$$

(17d) $$\mu =f_{x}\;\cdot \;\nu_{1}.$$

(17e) $$\mu_{d}=\mu_{d,\min}+\mu_{d,\mathrm{add}}\;\cdot \;\frac{C}{C+K_{d,C}}.$$

(17f) $$\nu_{1}=\nu_{1,\max}\;\cdot \;\frac{A}{A+K_{M,A}}.$$

(17g) $$\nu_{3}^{-}=\nu_{3,\max}^{-}\;\cdot \;\frac{C}{C+K_{M,C}}\;\cdot \;\frac{K_{I,A}}{A+K_{I,A}}.$$

(17h) $$v_{3}^{+}=0,\;\text{if}\;v_{1}<v_{\left(2,max\right)}.$$

(17i) $$v_{3}^{+}=v_{1}-v_{\left(2,max\right)},\text{if},v_{1}\geq v_{\left(2,max\right)}.$$

(17j) $$\nu_{3}=\nu_{3}^{+}-\nu_{3}^{-}.$$

(17k) $$\nu_{2}=\nu_{1}-\nu_{3}.$$

(17l) $$\frac{\mathrm{dV}}{\mathrm{dt}}={\dot{V}}_{\mathrm{Feed}}.$$

(17m) $$D=\frac{{\dot{V}}_{\mathrm{Feed}}}{V}.$$

**Table 4:** Parameter values, initial conditions, and inputs used for the generation of artificial fed‐batch data

Parameter name	value	unit
$A_{\mathrm{in}}$	300	mmol/L
$f_{x}$	0.1	g/mmol
$\mu_{d,\min}$	0.01	1/hr
$\mu_{d,\mathrm{add}}$	0.025	1/hr
$K_{d,C}$	15	mmol/L
$\nu_{1,\max}$	1.5	mmol/hr/g
$K_{M,A}$	40	mmol/L
$\nu_{3,\max}^{-}$	0.25	mmol/hr/g
$K_{M,C}$	10	mmol/L
$K_{I,A}$	5	mmol/L
$\nu_{2,\max}$	0.5	mmol/hr/g
A(t=0)	100	mmol/L
C(t=0)	0	mmol/L
$X_{v}\left(t=0\right)$	0.1	g/L
V(t=0)	1	L
${\dot{V}}_{\mathrm{Feed}}\left(t\in \left\{\left[0,80),[85,120),[125,140),[145,200\right]\right\}\right)$	0	L/hr
${\dot{V}}_{\mathrm{Feed}}\left(t\in \left\{\left[0,80),[80,85),[120,125),[140,145\right)\right\}\right)$	0.05	L/hr

Given initial conditions and a feeding profile ${\dot{V}}_{\mathrm{Feed}}(t)$, the profile of the concentrations A(t), C(t), and $X_{v}(t)$ as well as the reaction rate vector $\underline{\nu }(t)$ can be simulated using an ODE solver. In this case study, the implicit Matlab® solver ode15s was used.

The network can be decomposed into EM. The internal and external stoichiometric matrices N and P are:

(18) $$N=\left[\begin{array}{ccc} 1 & -1 & -1 \end{array}\right]⟵B.$$

(19) $$P=\left[\begin{array}{ccc} 0 & 0.1 & 0\\ -1 & 0 & 0\\ 0 & 0 & 1 \end{array}\right]\begin{array}{c} ⟵X_{v}\\ ⟵A\\ ⟵C \end{array}.$$

The conversion factor $f_{x}=0.1$ (cf. Equation (17d)) is included in the P matrix. The EM matrix E was calculated with metatool (Pfeiffer et al., 1999) from N (with reaction 1 and reaction 2 being irreversible):

(20) $$E=\left[\begin{array}{ccc} 0 & 1 & 1\\ 1 & 0 & 1\\ -1 & 1 & 0 \end{array}\right].$$

Using Equations (1) and (8), the set of differential equations describing the concentrations of components in the medium is:

(21) $$\frac{d}{\mathrm{dt}}\left[\begin{array}{c} X_{v}\\ A\\ C \end{array}\right]=P\;\cdot \;E\;\cdot \;\left[\begin{array}{c} r_{1}\left(t\right)\\ r_{2}\left(t\right)\\ r_{3}\left(t\right) \end{array}\right]\;\cdot \;X_{v}.$$

The matrix P⋅E contains the stoichiometry of all EM wrt. the external concentrations. All columns are scaled, such that the norm equals one. The death rate is added as a fourth column. Equation (21) is then extended to:

(22) $$\frac{d}{\mathrm{dt}}\left[\begin{array}{c} X_{v}\\ A\\ C \end{array}\right]=\left[\begin{array}{cccc} 0.0995 & 0 & 0.0995 & -1\\ 0 & -0.7071 & 0.9950 & 0\\ -0.995 & 0.7071 & 0 & 0 \end{array}\right]\;\cdot \;\left[\begin{array}{c} r_{1}\left(t\right)\\ r_{2}\left(t\right)\\ r_{3}\left(t\right)\\ \mu_{d}\left(t\right) \end{array}\right]\;\cdot \;X_{v}.$$

The cell‐specific reaction rates of these EM, ${\underline{r}}^{\mathrm{true}}\left(t\right)$ can be calculated from the vector $\underline{\nu }\left(t\right)$ using Equation (8).

## APPENDIX D EM ANALYSIS USING NOISY CONCENTRATION DATA

### Estimation of specific uptake and secretion rates

The estimation is carried out according to Equation (23), where the derivative dC/dt is obtained numerically using central differences from interpolated concentration measurements:

(23) $$q_{i}\left(t\right)=\frac{{\frac{dC_{i}}{\mathrm{dt}}|}_{t}-D\left(t\right)\;\cdot \;\left(C_{i,\mathrm{in}}-C_{i}\left(t\right)\right)}{X_{v}\left(t\right)}$$

To counteract the effect of measurement errors, the interpolation of concentration measurements is usually smoothed. We compare three different interpolation settings with splines, using the MATLAB® function spaps with different tolerance settings (0, $15\;\cdot \;{\bar{c}}_{i}$, $50\;\cdot \;{\bar{c}}_{i}$). The resulting rates $q_{i}(t)$ are then filtered with a moving average filter (MA) using either 30 or 90 values (where each value accounts for 1 hr of process time).

### Estimation of EM reaction rates using specific uptake‐ and secretion rates

The reaction rates of the EM $\underline{r}$ of each time‐point are obtained from $\underline{q}$ either with the method of Poolman et al. (2004) (Equation (24)) or Soons et al. (2011) (Equation (25)).

(24) $$\underline{r}={\left(P\;\cdot \;E\right)}^{\#}\;\cdot \;\underline{q}$$

(25) $$\min_{\underline{r}}\left({\left\|P\;\cdot \;E\;\cdot \;\underline{r}-\underline{q}\right\|}_{2}^{2}\right)$$

The matrices P and E are built from the metabolic network (cf. appendix: generation of artificial fed‐batch data) and # denotes the Moore‐Penrose pseudo‐inverse.

### DMFA for EM

Before the data was analyzed, *pseudo‐batch* data was calculated from the fed‐batch data by eliminating feeding influences.

The tuning of the *DMFA for EM* method is carried out by using cross‐validation. Here, the estimation problem (11) was solved with a different number of inflection time‐points and regularization coefficients α. A few randomly selected blocks of measurements were excluded from the estimation. The mean *SSR* value of this test‐sets was then analyzed and used as a measure of an appropriate selection of the number of inflection time‐points and α. It was found that 5 inflection points with a regularization parameter of α≈10 are the optimal choice in this case study.

### Comparison of methods

To evaluate the effect of random measurement noise, it is assumed that the measurements are subject to Gaussian noise with zero mean and a standard deviation which is dependent on the magnitude of the measurements (such that the width of the 95% confidence interval is, e.g., 5% of the magnitude of the measurements). The sampling and the estimations were repeated 100 times at different levels of noise.
